# IRESeek: structure-informed deep learning method for accurate identification of internal ribosome entry sites in circular RNAs

**DOI:** 10.1093/nargab/lqaf210

**Published:** 2025-12-31

**Authors:** Feng Zhang, Heqin Zhu, Jiayin Gao, Jie Hu, Ke Chen, Shaohua Kevin Zhou, Peng Xiong

**Affiliations:** School of Biomedical Engineering, Division of Life Sciences and Medicine, University of Science and Technology of China (USTC), Anhui 230026, China; Suzhou Institute for Advance Research, USTC, Jiangsu 215123, China; School of Biomedical Engineering, Division of Life Sciences and Medicine, University of Science and Technology of China (USTC), Anhui 230026, China; Suzhou Institute for Advance Research, USTC, Jiangsu 215123, China; Center for Medical Imaging, Robotics, Analytic Computing & Learning (MIRACLE), Suzhou Institute for Advance Research, USTC, Jiangsu 215123, China; School of Biomedical Engineering, Division of Life Sciences and Medicine, University of Science and Technology of China (USTC), Anhui 230026, China; Suzhou Institute for Advance Research, USTC, Jiangsu 215123, China; School of Biomedical Engineering, Division of Life Sciences and Medicine, University of Science and Technology of China (USTC), Anhui 230026, China; Suzhou Institute for Advance Research, USTC, Jiangsu 215123, China; School of Biomedical Engineering, Division of Life Sciences and Medicine, University of Science and Technology of China (USTC), Anhui 230026, China; Suzhou Institute for Advance Research, USTC, Jiangsu 215123, China; School of Biomedical Engineering, Division of Life Sciences and Medicine, University of Science and Technology of China (USTC), Anhui 230026, China; Suzhou Institute for Advance Research, USTC, Jiangsu 215123, China; Center for Medical Imaging, Robotics, Analytic Computing & Learning (MIRACLE), Suzhou Institute for Advance Research, USTC, Jiangsu 215123, China; Jiangsu Provincial Key Laboratory of Multimodal Digital Twin Technology, Jiangsu 215123, China; State Key Laboratory of Precision and Intelligent Chemistry, USTC, Anhui 230026, China; School of Biomedical Engineering, Division of Life Sciences and Medicine, University of Science and Technology of China (USTC), Anhui 230026, China; Suzhou Institute for Advance Research, USTC, Jiangsu 215123, China

## Abstract

The internal ribosome entry site (IRES) is a special type of RNA *cis*-acting element that can initiate translation independently of the 5′ cap structure and is widely found in viral RNAs and eukaryotic messenger RNAs. In recent years, an increasing number of studies have revealed that IRES elements also exist in circular RNAs (circRNAs) and mediate their translation. CircRNAs exhibit high stability and tissue specificity, playing critical roles in various physiological and pathological processes. Their coding potential provides important clues for the discovery of novel functional proteins. However, due to the nonlinear structure of circRNAs and the complexity of IRES-mediated regulatory mechanisms, accurately identifying IRES elements within circRNAs remains a significant challenge. Here, we propose IRESeek, a dual-branch deep learning framework for highly accurate detection of IRES elements in circRNA, which utilizes transformer for RNA sequence modeling and graph convolutional network for RNA structural guidance. To grasp the structural patterns of circRNAs, IRESeek employs physical-based thermodynamic energy of RNA secondary structure—base pair motif energy and the base pair probability as guidance structural characteristics to incorporate with RNA sequence, enabling comprehensive joint learning of RNA sequence and base pair interactions.

## Graphical Abstract

**Graphical Abstract:**
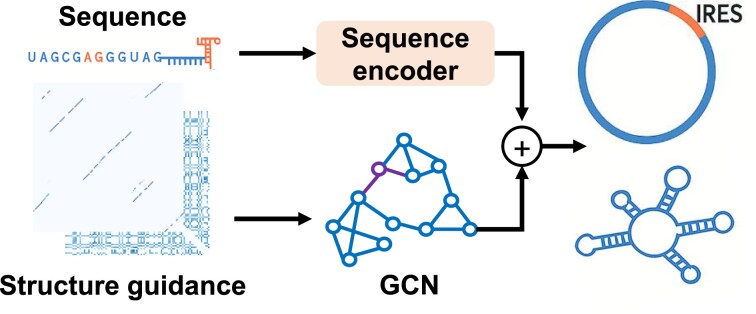

## Introduction

Circular RNAs (circRNAs) are covalently closed circRNA molecules distinguished by their topological stability and enhanced nuclease resistance compared to linear RNAs [1]. Notably, emerging evidence challenges the conventional noncoding paradigm of circRNAs, revealing their capacity for cap-independent translation initiation through internal ribosome entry site (IRES) elements despite lacking canonical 5′ cap structures [2–4]. These discoveries suggest that circRNAs play vital roles in multiple biological procedures, such as protein coding [5–8], regulation, and cancer diseases [9, 10].

The mechanism of circRNAs translation is different from conventional 5′ cap-dependent initiation mechanisms (including synthetic analogs like ARCA (anti-reversed cap) [11]), in which IRES-mediated translation represents an evolutionarily conserved strategy observed across viral genomes and eukaryotic cells [12, 13]. Typical IRES elements span 150–250 nucleotides and mediate in diverse cellular activities, including cell mitosis [14], modulation of proliferative signaling cascades [15], execution of programmed cell death pathways [16], and tumor suppression [17], reflecting functionalities in exploiting genetic therapies. In comparison with linear RNA IRESs, circRNA IRESs are more stable and structured with lower free energy, which directs to a promising future in RNA therapeutic targets, RNA therapeutics, and messenger RNA vaccines for cirRNAs [18]. Therefore, identifying IRES becomes a pivotal step in developing RNA-centric therapeutics, with profound implications for targeted cancer interventions and gene therapy innovations [19, 20].

Experimental verification of IRES, such as constructing expression vectors (synthetic bicistronic assays) [21], is time-consuming, resource-costing, and easily affected by various factors. As an alternative, with the building of IRES databases [22–24], more and more computational data-driven methods have been developed to identify IRES in a fast, cost-efficient, and accurate way. In the early time, IRSS [25] and VIPS [26] developed elaborately-designed procedures to assess the score and identify IRES structure in viral RNA sequences by utilizing RNALfold in ViennaRNA package [27] and RNA Align program [28] for predicting RNA secondary structure and comparing secondary structures, respectively. IRSS and VIPS are limited in viral RNA sequences and cannot be available now. Later, a variety of machine learning methods were applied to identify IRES and have made much progress in detecting accuracy [29–31]. For instance, IRESpy [31] employs a robust XGBoost-based machine learning framework that analyzes length-independent global *k*-mer features, effectively overcoming the limitations of conventional tools like VIPS [26] and IRESPred [29] that rely on length-dependent feature extraction strategies. Recent advances in deep learning architectures, exemplified by DeepIRES [32] and DeepCIP [33], have revolutionized the modeling of biological systems through diverse neural network paradigms. Modern architectures encompassing transformer networks [34], graph neural networks (GNNs) [35], and long short-term memory (LSTM) units [36] demonstrate excellent capacity in extracting discriminative features from RNA sequences.

Compared to sole primary sequence as input information, integrating RNA structural information into deep learning methods can provide more insights into ribosome recruitment and structural interactions between ribosome and IRES *trans*-acting factors [14, 37, 38]. Current methodologies harness the benefit of RNA secondary structures in naive modes. For instance, some methods [29, 39] utilize handcrafted structural features such as the number of predicted hairpin, bulge, and internal loops, statistic lengths of stem and loop to enrich the input structural information, while other methods like IRESpy [31, 33] make use of the estimated thermodynamic energy. However, IRESpy [31] found that the minimum free energy had little help in improving the model performances. Even state-of-the-art frameworks like DeepCIP [33] demonstrate critical limitations: their reliance exclusively on base pair probability (BPP) matrix derived from ViennaRNA [27, 40] is limited and insufficient for covering the whole space of base pair interactions. Furthermore, the absence of publicly available implementation and reproducible training code of DeepCIP fundamentally hinders methodological advancements and comparative analysis.

To bridge these technological gaps, we present IRESeek, an open-source effective deep-learning framework for IRES identification in circRNAs. Our contributions can be categorized into two parts: (i) IRESeek enables seamless integration and establishes implicit correlations between RNA sequences and structural information, including BPP [27, 40] and base pair motif energy (BPE) [41]. (ii) To deal with imbalanced datasets, IRESeek employs oversampling and undersampling strategies for training high-precision and high-recall models, respectively, and further ensemble them through soft voting for robust and versatile IRES identification.

## Materials and methods

### Dataset preparation

We adopt a widely used benchmark dataset for evaluating the performances of IRESeek and comparing it with other IRES prediction methods. The dataset used in this study is derived from Refs. [33, 42, 43]. This dataset is originally employed by high-throughput screening [42] for quantifying the IRES activity of synthetic oligonucleotide, using the oligonucleotide library from Ref. [43]. Specifically, this dataset consists of 4531 circRNA IRES (positive samples) and 9616 circRNA non-IRES (negative samples), with a uniform length of 174 nucleotides due to the artificially constructed oligonucleotide library. Zhou *et al.* [33] partition this dataset into a training set (3949 positive samples and 9034 negative samples) and a testing set (582 positive samples and 582 negative samples) for developing deep learning models (see Supplementary Table S1). Therefore, the training set contains imbalanced samples with a positive:negative ratio of 4:9, which should be dealt with to avoid model bias when training deep learning models.

### The architecture of IRESeek

**Figure 1.**
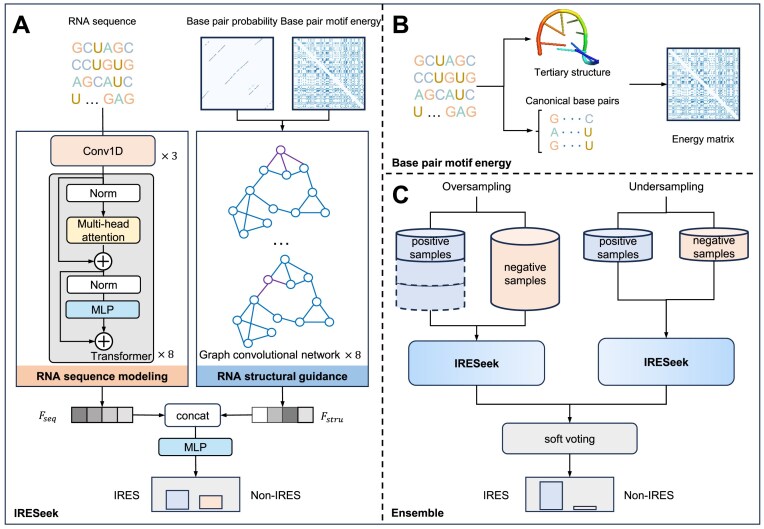
Overview of our IRESeek approach for IRES classification in circRNA. (**A**) IRESeek takes a dual-stream framework by employing 1D convolution, transformer network, and GCN for parallel modeling of RNA sequence and structural information, including BPP and BPE, which are further gathered as concatenated sequence–structure features and processed by multiple perceptrons to make the final prediction. (**B**) The detailed process for obtaining BPE of an arbitrary RNA sequence. (**C**) The ensemble model of IRESeek models that are learned on balanced oversampling and undersampling datasets.

As Fig. 1A demonstrates, IRESeek takes a dual-branch architecture with data flows of RNA sequence information and RNA structural guidance, respectively. Given an input sequence embedding $\mathbf {X}=\lbrace x_1,x_2,\ldots , x_i, \ldots , x_L\rbrace \in R^{L\times 4}$, where $x_i$ denotes the th embedding vector, we obtain structural matrices of base pair probability $M_{\rm p}$ (generated by RNAfold in ViennaRNA software package version 2.6.4 [27, 40]) and base pair motif energy $M_{\rm e}$ (generated by BPfold version 0.2.0 [41]), respectively. We utilize 1D convolution blocks and transformer blocks to build RNA sequence features $F_\text{seq}$, while we harness graph convolutional network (GCN) to capture structural features $F_\text{stru}$ ($M_{\rm p}$ and $M_{\rm e}$ are processed separately by GCN to learn structural features, and the resulting embeddings are concatenated to form the overall structural feature). The output classification scores $\mathbf {\tilde{Y}}$ of IRESeek are generated by applying multiple linear perceptron (MLP) [44] to the concatenation of $F_\text{seq}$ and $F_\text{stru}$, which can be formulated as follows:


(1) \begin{eqnarray*}
\mathbf {\tilde{Y}}= \mathrm{MLP}([F_\text{seq}:F_\text{stru}]).
\end{eqnarray*}

Furthermore, as Fig. 1C shows, to improve the robustness and stability of IRESeek trained on the imbalanced dataset (3949 positive samples and 9049 negative samples), we make soft voting of the predicted scores of IRESeek trained by oversampling and undersampling strategies. The following two subsections, RNA sequence modeling and RNA structural guidance, make detailed descriptions of the RNA sequence modeling and RNA structural guidance.

### RNA sequence modeling

As for an input RNA sequence of length $L$, firstly, we make the following preprocesses: (i) converting lowercase to uppercase and (ii) converting unknown characters to “A/U” or related paring bases. Next, each nucleotide in the sequence (A, U, T, G, and C) was encoded using one-hot encoding, with A represented as [1, 0, 0, 0], U and T as [0, 0, 0, 1], G as [0, 1, 0, 0], and C as [0, 0, 1, 0], with each nucleotide as a sequence token, obtaining an encoded input feature vector in shape of $L\times 4$, which will be fed into following neural networks for modeling long dependencies of RNA sequence.

As Fig. 1A shows, on the data flow branch of RNA sequence, we employ cascaded 1D convolution blocks [45] and transformer [34] blocks to extract representative features of RNA sequences in an effective and efficient way. Unlike BiLSTM layers (Bi-directional Long Short-Term Memory) [46], 1D convolutions are fast and lightweight to process sequential features with a large perceptive field, while transformer is much more suitable for molecular sequences that are greatly dependent on the previous and following contextual information. As Fig. 1B shows, we set three 1D convolutions with kernel size being $5, 3, 3$ to initially process embedding of RNA sequence $X=\lbrace x_1,x_2,\ldots , x_i, \ldots , x_L\rbrace \in R^{L\times 4}$, where each convolution operation is followed with batch norm layer and ReLU activation operation. The output feature $\mathbf {S}$ is obtained after applying these cascaded sequential convolution blocks. The procedures of convolution blocks can be formulated as follows:


(2) \begin{eqnarray*}
\begin{aligned} S_i &= \mathrm{ReLU}(\mathrm{BatchNorm{1D}}_{\theta ^{(i)}}(\mathrm{Conv{1D}}_{\theta ^{(i)}}(S_{i-1}))),\\S &= S_3, S_0 = X,\\\end{aligned}
\end{eqnarray*}

where $i=1, 2, 3$ is the th convolution block with its parameter $\theta ^{(i)}$.

Subsequently, the processed sequential features $S$ will be passed to transformer blocks for capturing the relationship of nucleotides and learning the contextual information of each base nucleotide to further model the global dependencies and position-aware information of the whole sequence. Transformer blocks are based on attention mechanism, which is effective for parallel computing and global modeling. Each transformer block contains a multihead self-attention module (MHSA) and a feed-forward network (FFN). Before processing, the input feature will be added with a positional embedding for position-aware modeling. Furthermore, there are layer normalization and residual operations before MHSA and FFN layers. These operations can be formulated as follows:


(3) \begin{eqnarray*}
\begin{aligned} H^{(1)} &= S,\\\tilde{H}^{(i)} &= \mathrm{LN}_{\theta _i}(H^{(i)}),\\H_Q^{(i)} &= {Q}_{\theta ^{(i)}}(\tilde{H}^{(i)}),\\H_K^{(i)} &= \mathrm{K}_{\theta ^{(i)}}(\tilde{H}^{(i)}),\\H_V^{(i)} &={V}_{\theta ^{(i)}}(\tilde{H}^{(i)}),\\H_{\text{MHSA}}^{(i)} &= \mathrm{softmax}\left(\frac{H_Q^{(i)} {H_K^{(i)}}^T}{\sqrt{d}}H_V^{(i)}\right),\\\tilde{H}_{\text{MHSA}}^{(i)} &= \mathrm{LN}_{\theta ^{(i)}}(H_{\text{MHSA}}^{(i)} + \tilde{H}^{(i)}),\\H^{(i+1)} &= \tilde{H}_{\text{MHSA}}^{(i)} + \mathrm{FFN}_{\theta ^{(i)}}(\tilde{H}^{(i)}_{\text{MHSA}})),\\F_\text{seq} &= H^{(8)}, \end{aligned}
\end{eqnarray*}

where $i=1, 2, \ldots , N-1$ with $N$ being the number of transformer blocks, $\theta ^{(i)}$ denotes the learning parameters of the th neural network module, and $d$ is the hidden dimension for scaling the attention matrix. We set $N=8$ for transformer blocks.

RNA secondary structure mainly depends on the complex interactions between bases, which are hard to represent through traditional handcrafted features. Here, we harness the combined convolution and transformer neural networks for building long-range sequential dependencies, aiming at providing representative features extracted from the RNA sequence for accurate IRES classification.

### RNA structural guidance

The information provided by RNA sequence only is limited, which makes it hard for deep learning models to accurately classify whether there is IRES in a circRNA. Here, we introduce RNA structural information to guide the classification of IRES. Specifically, we use two kinds of structural information for enhanced input features:

- BPE that estimates the thermodynamic and statistical energy potential of a possible base pair in the RNA sequence by modeling the local structure of three neighboring bases upstream and downstream of the base pair, which is a matrix denoted as $M_e$ in the shape of $L\times L$ generated by BPfold version 0.2.0 [41]. The element of the BPE matrix is within the range of $[-1, 1]$. The BRIQ energy score represents a composite energy metric that integrates physical energy calculations derived from density functional theory (DFT) with statistical energy terms calibrated via quantum mechanical principles. This composite score provides a balanced assessment of thermodynamic energy, striking a compromise between computational efficiency and accuracy. As demonstrated in the BPfold [41] framework, individual energy scores corresponding to each base pair motif undergo normalization based on both their sequence length and motif classification.
- BPP that provides the information of interactions between bases, which is a matrix denoted as $M_{\rm p}$ in the shape of $L\times L$ generated by RNAfold in ViennaRNA software package version 2.6.4 [27, 40]. The element of BPP is within the range of $[0, 1]$.

After collecting structural information from RNAfold and BPfold tools, we construct two graphs $G_{\rm p}$ and $G_{\rm e}$ for BPP matrix $M_{\rm p}$ and BPE matrix $M_{\rm e}$, respectively. In each graph, the nucleotide base is a graph node, and the nonzero element of the structural information matrix is stored in an undirected edge between the two nodes. Each RNA sequence together with its structural connects can be formulated as follows:


(4) \begin{eqnarray*}
\begin{aligned} G_{\rm p} &= (V, E_{\rm p)},\\G_{\rm e} & = (V, E_{\rm e}),\\\end{aligned}
\end{eqnarray*}

where $V$ is the set of nucleotide bases and $E$ is the set of edges that store the structural information.

After constructing the structural graphs, we utilize GNN [35] to learn the relationship of adjacent nodes, which is different from previous methods that convert structural information into one-hot matrices and employ convolutional networks to process them. GNN is capable of directly processing structural data and is effective in the communication of nodes. GNN is learned iteratively by the message passing mechanism, which makes each node aggregate information from adjacent nodes and capture contextual information from the structural graph. Here, we use GCN [47] as the backbone model. In the iteration process, we apply a symmetrically normalized adjacency matrix to node features for linear and nonlinear transformations. After processing by GCN layers, we apply maximum pooling to the features to obtain the structural aggregated feature for the classification of IRES. These procedures can be formulated as follows:


(5) \begin{eqnarray*}
\begin{aligned} F_{\text{stru}}^{(i)} &= \sigma ( \tilde{D}^{-\frac{1}{2}}\tilde{M}\tilde{D}^{-\frac{1}{2}}F_{\text{stru}}^{(i-1)}W^{(i)}),\quad i=1,2,\ldots , 8,\\F_{\text{stru}} &= \mathrm{MAXPOOL}(F_{\text{stru}}^{8}), \end{aligned}
\end{eqnarray*}

where $\tilde{M} = M+I$ denotes the structural matrix plus of unit matrix, $\tilde{D}$ denotes the degree matrix of $\tilde{M}$, $W^{(i)}$ denotes the weight matrix of the th neural network layer, $F_{\text{stru}}^{(i)}$ denotes the structural feature of the th neural network layer, and $\sigma (\cdot )$ denotes nonlinear activation function. $\tilde{D}^{-\frac{1}{2}}\tilde{M}\tilde{D}^{-\frac{1}{2}}$ performs normalization to adjacency matrix $M$ by considering both connected nodes (base pair).

### Strategy for imbalanced classification

The training dataset contains 3949 positive samples and 9049 negative samples, which is imbalanced and may lead to model classification bias. To tackle this issue, we resort to ensemble models that learn about oversampling dataset and undersampling dataset. As Fig. 1C demonstrates, specifically, we construct two balanced datasets by duplicating two times of the positive samples for oversampling and randomly selecting one-third negative samples for undersampling. We train IRESeek on each constructed dataset and obtain two models, and the aggregated output scores are obtained by soft voting of the predictions of the two models, which makes IRESeek more robust and stable.

### Learning objectives and evaluation metrics

At the training stage, given an input RNA sequence and ground truth label $\mathbf {Y}$, IRESeek outputs the prediction scores $\mathbf {\tilde{Y}}$ of positive and negative classes. Therefore, we adopt a cross-entropy loss (CE) $\mathcal {L}$ to optimize the learning of IRESeek:


(6) \begin{eqnarray*}
\begin{aligned} \mathbf {\tilde{Y}^\text{prob}} &= \mathrm{softmax}(\mathbf {\tilde{Y}}),\\\mathcal {L} &= \mathrm{CE} (\mathbf {\tilde{Y}^\text{prob}},\mathbf {Y}), \\&= - \frac{1}{N}\sum _{n=1}^N[y_n {\rm log}(\tilde{y}^\text{prob}_n) + (1-y_n) {\rm log}(1-\tilde{y}^\text{prob}_n)]. \end{aligned}
\end{eqnarray*}

At the testing stage, we use the following widely used evaluation metrics to comprehensively assess the performance of the proposed IRESeek and other IRES identification methods, including accuracy (ACC), Precision (P), Recall (R), F1 score (F1), and Matthews correlation coefficient (MCC). Accuracy is simple and direct, but sensitive to imbalanced cases. Precision focuses on reducing false positive samples, while Recall focuses on reducing false negative samples. F1 score harnesses Precision and Recall for robust evaluation of imbalanced cases. MCC measures all kinds of classification results. Each of these metrics is defined as follows:


(7) \begin{eqnarray*}
\begin{aligned} \text{ACC} &= \frac{\text{TP}+\text{TN}}{\text{TP}+\text{TN}+\text{FP}+\text{FN}},\\\text{P} &= \frac{\text{TP}}{\text{TP}+\text{FN}},\\\text{R} &= \frac{\text{TP}}{\text{TP}+\text{FP}},\\\text{F1} &= \frac{2\times \text{P}\times \text{R}}{\text{P}+\text{R}},\\\text{MCC} &= \frac{\text{TP}\times \text{TN}-\text{FP}\times \text{FN}}{\sqrt{(\text{TP}+\text{FN})(\text{TP}+\text{FP})(\text{TN}+\text{FP})(\text{TN}+\text{FN})}}, \end{aligned}
\end{eqnarray*}

where TP, TN, FP, and FN represent true positive, true negative, false positive, and false negative, respectively.

- TP is the number of IRES sequences correctly predicted as IRES;
- TN is the number of non-IRES sequences correctly predicted as non-IRES;
- FP is the number of non-IRES sequences incorrectly predicted as IRES; and
- FNis the number of IRES sequences incorrectly predicted as non-IRES.

## Results

### Effectiveness of RNA structural guidance

To comprehensively assess the effectiveness of the key components of IRESeek, i.e. structural guidance of BPE and BPP, we conduct ablation studies to demonstrate the performances of IRESeek with different input information. (i) seq+BPE+BPP: the final version of IRESeek, including RNA sequence, BPE, and BPP; (ii) seq+BPE: including RNA sequence and BPE; (iii) seq+BPP: including RNA sequence and BPP; and (iv) seq: only RNA sequence. In this experiment, we train IRESeek under these four different configurations, adopting the ensemble models of combining oversampling and undersampling strategies.

**Figure 2.**
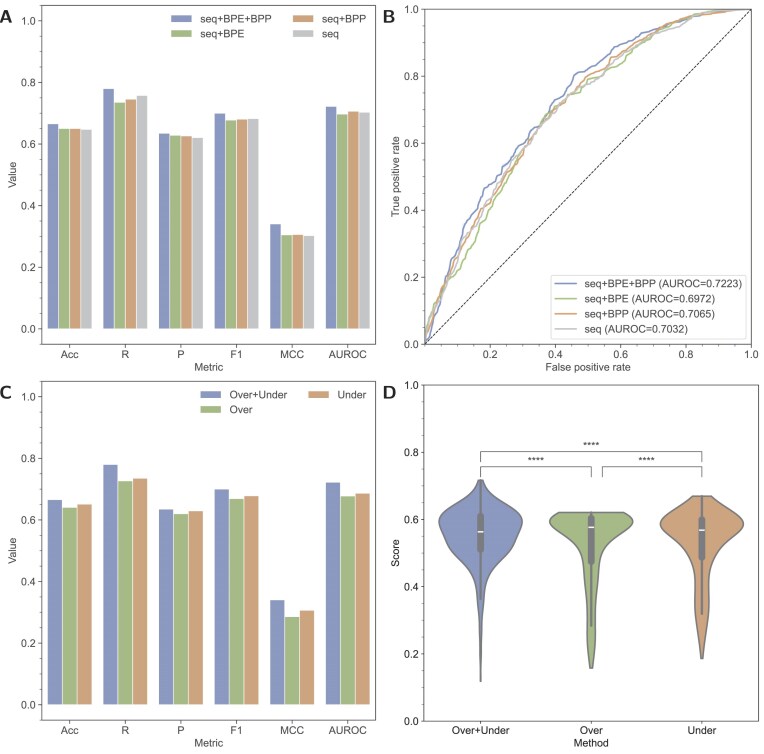
Ablation study of IRESeek on structural guidance and sampling strategy. (**A**) Ablation study of IRESeek on structural guidance of BPE and base pair motif probability (BPP): (i) seq+BPE+BPP; (ii) seq+BPE; (iii) seq+BPP; and (iv) seq. Positive samples (*n*= 582 RNAs) and negative samples (*n*= 582 RNAs) are presented as bars under six metrics: accuracy (ACC), recall (R), precision (P), F1 score (F1), MCC, and AUROC. (**B**) ROC curve visualization of predicted scores in panel (A). (**C**) Ablation study of IRESeek on sampling strategies for imbalanced classification: (i) Over+Under: ensemble of oversampling and undersampling models; (ii) Over: oversampling; (iii) Under: undersampling. Positive samples (*n* = 582 RNAs) and negative samples (*n* = 582 RNAs) are presented as bars under above six metrics. (**D**) Violin visualization of predicted scores of positive samples (*n*= 582 RNAs) in panel (C) with statistical significance analysis (ns: $P > .05$; *: $P \le .05$; **: $P \le .01$; ***: $P \le .001$; ^****^: $P \le .0001$).

By combining and configuring the different elements mentioned above, systematic comparisons and analysis are conducted and visualized in Fig. 2 and other head-to-head comparisons and metrics presented in Supplementary Figs S1–S3. As Fig. 2A and Supplementary Table S2 show, by employing BPE and BPP structural guidance, IRESeek achieves the highest performances under all kinds of metrics, especially large improvements in recall, which indicates that the specific structure and base pair interactions of RNA sequence boost the identification of IRES. As Fig. 2B shows, seq+BPE+BPP obtains an area under ROC (AUROC) curve of 0.7223, much better than the second place (seq+BPP) of 0.7065, proving that BPE plays a vital role in improving the robustness of IRESeek by providing thermodynamic priors. In detail, the complete model (seq+BPE+BPP) is superior to other models under metrics of ACC, R, P, F1, MCC, and AUROC, with improvement ranges being 1.5%–1.8%, 2.3%–4.5%, 0.7%–1.4%, 1.8%–2.2%, 3.4%–3.7%, and 1.5%–3.2%, respectively. These results show that the structural guidance characteristics of BPE and BPP play an important role in improving the overall performance of the model, especially in improving the discrimination ability and generalization ability of the model.

### Effectiveness of model ensemble strategy

In addition, we further carry out an ablation study on the sampling strategies to verify the effectiveness of our proposed ensemble models that trained on different sampling strategies, i.e. the soft fusion of oversampling and undersampling model, comparing with the model using oversampling or undersampling alone. As shown in Fig. 2C and D and Supplementary Table S3, it is evident that the undersampling strategy is slightly better than the oversampling strategy under all metrics, which indicates that the redundant positive IRES sequences sampled by the oversampling strategy may lead the model to learn redundant patterns and hinder the further improvement of performances. However, when we combine the oversampling and undersampling strategies, harnessing both of their advantages in high precision and recall, respectively, the performances improve in a huge gap and are superior to the other two methods in terms of the six evaluation metrics, including ACC, R, P, F1, MCC, and AUROC. Specifically, the performance improvement range is as follows: ACC (increased by 1.5%–2.5%), R (increased by 4.5%–5.3%), P (increased by 0.6%–1.5%), F1 (increased by 2.2%–3%), MCC (increased by 3.3%–5.5%), and AUROC (increased by 3.5%–4.4%). These results highlight the robustness and stability of the integration strategy in dealing with imbalanced positive and negative IRES samples. Furthermore, we evaluate the advantages of the proposed method in identifying positive IRES samples by visualizing the predicted score distribution of positive samples in the test set in the form of a violin plot. As shown in Fig. 2D, compared with the baseline strategy, our method shows higher accuracy and stronger discrimination ability in distinguishing positive IRES samples, confirming the application potential and effectiveness of the integration strategy in imbalanced classification tasks.

### IRES identification performance of circular RNAs

**Figure 3.**
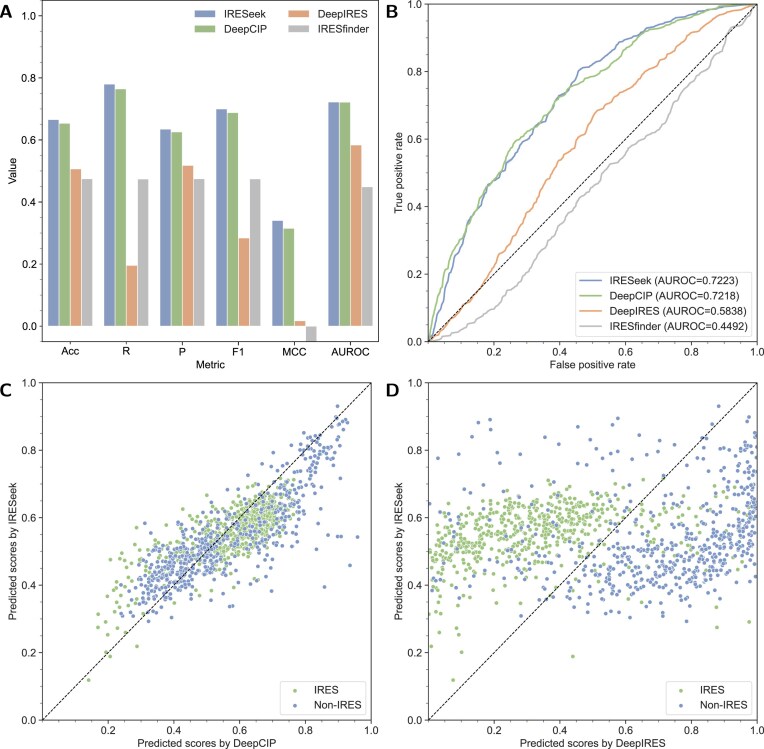
Performance comparison of IRESeek with three state-of-the-art IRES identification methods. (**A**) The performance comparison of DeepIRES, DeepCIP, and IRESfinder. Results are presented as bars under six metrics: accuracy (ACC), recall (R), precision (P), F1 score (F1), MCC, and AUROC. (**B**) Visualization of ROC curves and the metric of AUROC. (**C**) Head-to-head comparison of predicted scores between IRESeek and DeepCIP for IRES and non-IRES samples. (**D**) Head-to-head comparison of predicted scores between IRESeek and DeepIRES for IRES and non-IRES samples.

It is necessary to systematically assess the IRES identification performances of IRESeek by contrasting it with other state-of-the-art methods. Here, we carry out experiments to make performance comparisons with three deep learning-based methods, i.e. DeepCIP [33], DeepIRES [32], and IRESfinder [30] on the test dataset. As shown in Fig. 3A and Supplementary Table S4, quantitative analysis based on six classification evaluation indicators shows that IRESeek has significant advantages in overall performance: compared with existing methods, its ACC (increased by 1.2%–19.1%), R (increased by 1.5%–58.4%), P (increased by 1%–16%), F1 (increased by 1.2%–41.6%), and MCC (increased by 2.6%–39.1%) are significantly improved. DeepCIP achieves second place, whose performances are slightly lower than our proposed IRESeek under metrics like accuracy, recall, precision, F1 score, and AUROC. These results show that IRESeek not only performs well in the overall classification performance, but also effectively alleviates the widespread category imbalance problem in the IRES dataset by introducing the dynamic weight adjustment mechanism. We also evaluate the model’s discrimination capability using the AUROC metric, as illustrated in Fig. 3B. The results demonstrate that IRESeek achieves an AUROC of 0.7223, which is comparable with DeepCIP (0.7218), much better than DeepIRES (0.5838) and IRESfinder (0.4492), indicating that IRESeek has stronger discrimination ability in distinguishing IRES and non-IRES sequences.

To figure out the identification performance of each sample RNA sequence, we make head-to-head comparisons between IRESeek and other methods, and scatter the predicted score of each sample point of positive and negative IRES. As shown in Fig. 3C, IRESeek is comparable with DeepCIP in distinguishing IRES and non-IRES sequences. Considering that DeepIRES is mainly for the IRES prediction task of linear RNA, Fig. 3D shows the comparison results with IRESeek, which demonstrates that IRESeek has higher discrimination ability in identifying IRES and non-IRES sequences while DeepIRES tends to predict a high score value for negative samples and results in low identification precision. The above visual analysis results not only support the improvement of IRESeek in classification accuracy, but also reflect its advantages in prediction stability and reliability, especially in the task of identifying functional IRES elements in circRNA.

### Hyperparameter optimization

**Figure 4.**
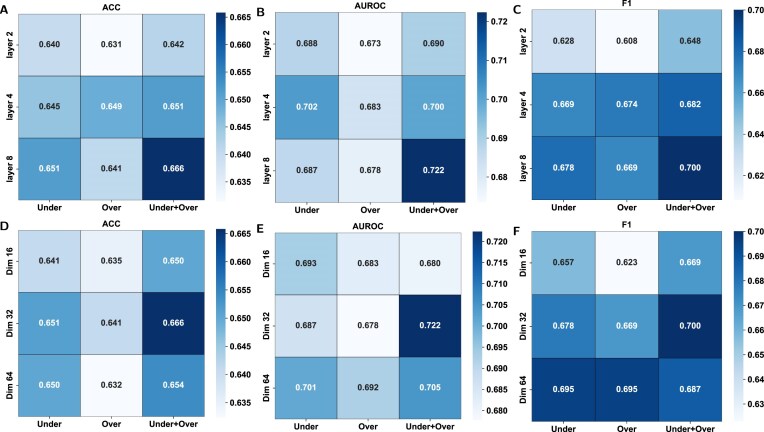
Visualization of hyperparameter optimization performance for different configurations of IRESeek. (**A**–**C**) The performance heatmaps of the number of GCN layers for structure guidance, together with sampling strategy, under metric accuracy, AUROC, and F1 score, respectively. (**D**–**F**) The performance heatmaps of the hidden layer feature dimension for sequence modeling, together with the sampling strategies, under metric accuracy, AUROC, and F1 score, respectively.

In this subsection, we first compared the performance of different sequence and structure model combinations in IRESeek (Supplementary Table S5). The results show that the combination of CNN + transformer with GCN (IRESeek) performs best across all metrics, demonstrating superior predictive capability. We then carry out a systematic analysis of the hyperparameter of IRESeek, including the hidden layer feature dimension for sequence modeling and the number of GCN layers for structure guidance, together with the sampling strategies. Considering that the model in this study is built through the integration of the oversampling model and undersampling model, we first classify the models, including the oversampling-only model, undersampling-only model, and integrated model integrating oversampling and undersampling strategies. In Fig. 4 (with other detailed metrics provided in Supplementary Tables S6–S9), we set three kinds of configurations of hyperparameter to determine the optimal hyperparameter configuration, so as to comprehensively analyze the impact of various factors on the final performance of IRESeek.

As the core component of extracting structural features, the configuration of the GCN module in IRESeek has an important impact on the performance of the model. In this study, we constructed GCN modules with different layers (including two layers, four layers, and eight layers) to explore the impact of different layer settings on the performance of various models. As demonstrated in Fig. 4A–C, with the increase of GCN layers, the overall performance of the model shows a steady improvement. Among them, the eight-layer GCN structure has achieved the best results under ACC, AUROC, and F1 metrics, respectively. In addition, among the three sampling strategies, the under+over combined sampling strategy is the most superior, which is significantly superior to the method of using undersampling or oversampling alone. Considering that more GCN layers and more complex model structures need more data and computational resources, we adopt the eight-layer GCN of IRESeek as a trade-off.

Similar to the GCN layer, we further analyze the impact of hidden feature dimensions on model performance. As shown in Fig. 4D–F, we systematically evaluated the performance of the model under different feature dimension settings (Dim = 16, 32, and 64) combined with three sampling strategies. The results show that consistent with the previous group of experiments, the under+over combined sampling strategy still shows the best performance in most configurations, especially in the setting of Dim = 32, the model achieves the performances under the three metrics. On the whole, the moderate feature dimension (Dim = 32) achieves a better balance between the ability of feature expression and the ability of model generalization.

### Interpretable visualization for circRNA IRES features

**Figure 5.**
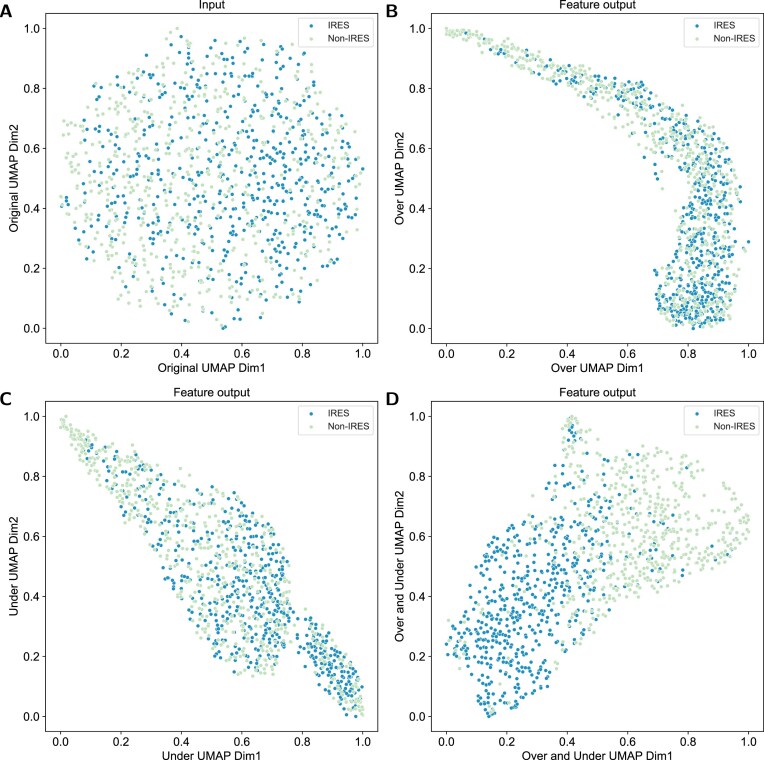
Visualization of Umap results for original data and different models. (**A**) Umap visualization of original input. (**B**) Umap visualization of the feature output of an oversampling model. (**C**) Umap visualization of the feature output of an undersampling model. (**D**) Umap visualization of the feature output of an ensemble model combining oversampling and undersampling.

Although predictive performance is essential, interpretability plays a crucial role in understanding how the model makes biologically meaningful decisions. Here, we conduct a visualization analysis to examine how well our proposed IRESeek captures discriminative features for IRES prediction. In this study, we utilize the widely used nonlinear dimensionality reduction method Umap [48] to visualize the original input features and the output features extracted from the IRESeek neural networks (other visualization methods are provided in Supplementary Figs S4 and  S5). As shown in Fig. 5A, the distributions of IRES and non-IRES samples in the original input features overlap in a big area, making it difficult to distinguish effectively. By conducting dimensionality reduction visualization on the features extracted by the oversampling model and the undersampling model, respectively, as shown in Fig. 5B and C, the results showed that the distribution of the two types of samples in the two models still overlapped significantly. In contrast, the feature representation extracted by IRESeek (over and under) shows a stronger discriminant ability (see Fig. 5D). The distribution of IRES and non-IRES samples is significantly more separated, the overlapping areas are significantly reduced, and the clustering is more compact. Furthermore, we applied the Integrated Gradients method [49] to the sequence feature extraction modules of each model to visualize the importance of *k*-mer sequences. As shown in Supplementary Fig. S6, the resulting consensus motifs predominantly exhibit AU-rich characteristics, which is highly consistent with previous research findings [50–52]. This further validates the potential of our model in uncovering biologically relevant features.

These results suggest that IRESeek is capable of automatically learning informative and discriminative representations that facilitate the accurate classification of IRES elements. This improved separability in the feature space also demonstrates the model’s ability to capture biologically relevant patterns, thereby enhancing its interpretability and potential utility in downstream biological analysis.

## Discussion

In this study, we propose IRESeek, aiming at integrating biological priors especially structural guidance into a deep learning method, to improve the identification performance of IRES in circRNAs. IRESeek combines BPE and BPP with graph-based structural modeling, which reflects the importance of introducing biological priors into neural networks and enables the model to capture features closer to the actual biological mechanism rather than relying solely on implicit sequence patterns. This structural guidance not only improves the classification performance, but also conforms to the headless dependent translation initiation mechanism mediated by IRES. Meanwhile, IRESeek adopts the strategy of combining oversampling and undersampling to effectively alleviate the deviation problem caused by imbalanced categories of IRES, which is particularly critical in the field of bioinformatics with a large number of negative samples and uneven quality, thus significantly enhancing the stability and generalization ability of the model under different data distributions. The ability of the model to distinguish IRES elements in a data-imbalanced setting suggests that our sampling strategies and structural-aware architectures can bridge the gap between model accuracy and biological interpretability.

Although IRESeek has demonstrated strong performance in our experiments, there remains room for further improvements. Firstly, the current training dataset mainly consists of artificially designed sequences with a fixed length of 174 nucleotides. However, in real biological systems, the lengths of IRES elements vary considerably. To enhance the model’s generalization ability and practical applicability, it is necessary to incorporate more experimentally validated IRES and non-IRES sequences of varying lengths, thereby constructing a more comprehensive and reliable training dataset. Secondly, future research may explore richer structural features, especially more complex secondary structure information as well as experimentally determined [53–56] and computational tertiary structural features characteristics [57–61], to further improve IRESeek’s prediction accuracy and interpretability in identifying such IRES elements.

In addition, integrating IRES information into the design of circRNA may provide novel strategies to optimize translation efficiency. Future studies could further investigate, through experimental, the potential applications of IRES in regulating circularization mechanisms, whether relying on natural pathways or via RNA circularization driven by specific binding of microRNAs [62].

## Conclusion

In this paper, we propose a deep learning framework, IRESeek, which incorporates physical-based thermodynamic energy of RNA secondary structure—BPE—to enhance the extraction of structural features. By combining both oversampling and undersampling strategies, IRESeek effectively addresses the data imbalance issue in circRNA IRES prediction tasks. The framework employs a GCN module to integrate structural information and leverages key hyperparameter optimization to achieve robust and accurate performance in distinguishing IRES from non-IRES sequences. Extensive experimental results demonstrate that the combined sampling strategy significantly improves the model’s generalization ability, while the well-balanced model architecture ensures an optimal trade-off between complexity and performance. Furthermore, visualization analysis indicates that the feature representations extracted by IRESeek exhibit stronger discriminative power and higher interpretability compared to other baseline models. In addition, the strong interpretability of IRESeek indicates its potential value in downstream experimental validation and hypothesis generation. As computational approaches become increasingly essential in life sciences, frameworks that effectively integrate biological priors with computational modeling will play an indispensable role in uncovering genomic functional mechanisms.

## Supplementary Material

lqaf210_Supplemental_File

## Data Availability

All datasets and codes used in this study are freely available at https://github.com/f-zhangf/IRESeek and https://doi.org/10.5281/zenodo.17734640.
