# Supplementary material for: IRESeek: structure-informed deep learning method for accurate identification of internal ribosome entry sites in circular RNAs
Source: NAR Genom Bioinform. 2025 Dec 31;7(4):lqaf210. doi: 10.1093/nargab/lqaf210 (PMC12754787; doi:10.1093/nargab/lqaf210)
Supplement: lqaf210_Supplemental_File [file lqaf210_supplemental_file.pdf]

Supplementary material for IRESeek: Structure-informed deep learning method for accurate identification of internal ribosome entry sites in circular mRNAs

Feng Zhang[ǂ1,2], Heqin Zhu[ǂ1,2,3], Jiayin Gao[1,2], Jie Hu[1,2], Ke Chen[1,2], Peng Xiong[1,2*], S. Kevin Zhou[1,2,3,4,5*]

[1]School of Biomedical Engineering, Division of Life Sciences and Medicine, University of Science and Technology of China (USTC), Hefei, Anhui, 230026, China.
[2]Suzhou Institute for Advanced Research, USTC, Suzhou, Jiangsu, 215123, China.
[3]Center for Medical Imaging, Robotics, Analytic Computing & Learning (MIRACLE), Suzhou Institute for Advance Research, USTC, Suzhou, Jiangsu, 215123, China.
[4]Jiangsu Provincial Key Laboratory of Multimodal Digital Twin Technology, Suzhou, Jiangsu, 215123, China.
[5]State Key Laboratory of Precision and Intelligent Chemistry, USTC, Hefei, Anhui, 230026, China.

*Corresponding author(s). E-mail(s): xiongxp@ustc.edu.cn; skevinzhou@ustc.edu.cn;

[1]Feng Zhang and Heqin Zhu contributed equally to this work.

1 Supplementary Figures

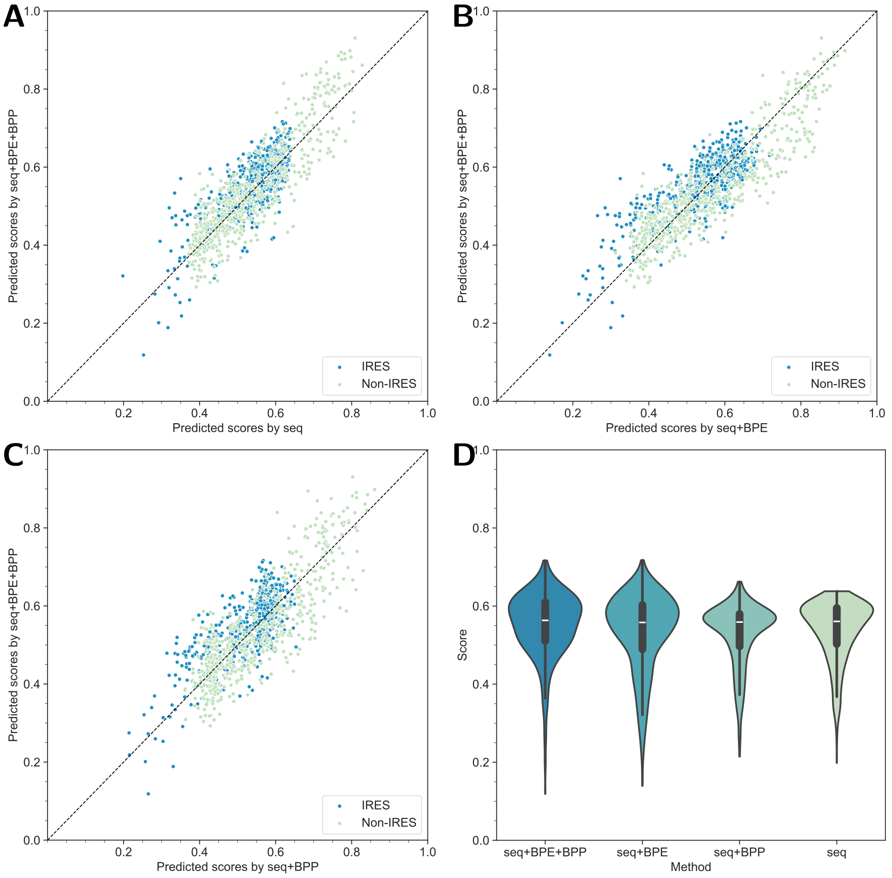

Supplementary Fig. 1: **Ablation study of IRESeek on structural guidance of base pair motif energy (BPE) and base pair motif probability (BPP).** **(A)** Head-to-head comparison of predicted scores between seq+BPE+BPP and seq for IRES and non-IRES samples.**(B)** Head-to-head comparison of predicted scores between seq+BPE+BPP and seq+BPE for IRES and non-IRES samples. **(C)** Head-to-head comparison of predicted scores between seq+BPE+BPP and seq+BPP for IRES and non-IRES samples. **(D)** Violin visualization of predicted scores of samples in seq, seq+BPP, seq+BPE, seq+BPP+BPE.

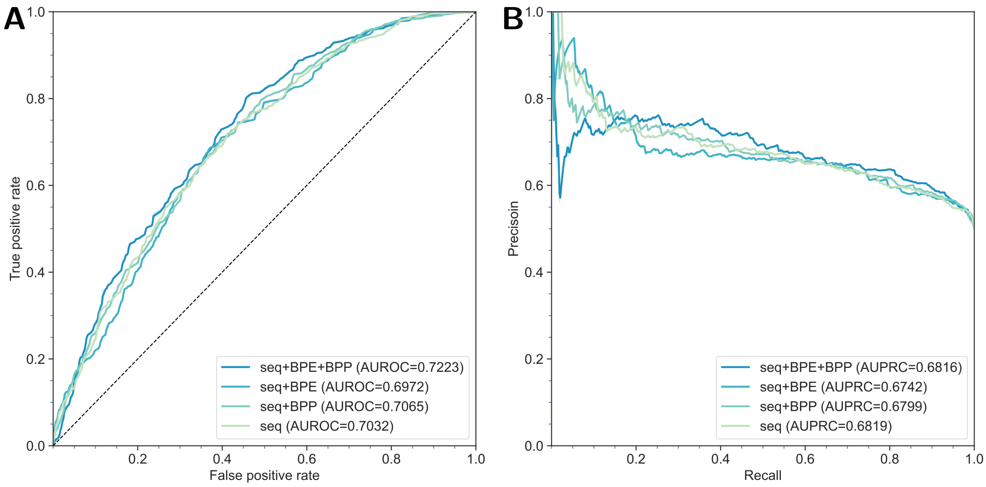

Supplementary Fig. 2: **Performance evaluation and comparison of different structural guidance including seq, seq+BPE, and seq+BPE+BPP: insights from Receiver Operating Characteristic (ROC) and Precision - Recall Curve Analysis.** **(A)**Receiver Operating Characteristic (ROC) Curves for seq, seq+BPP, seq+BPE, seq+BPP+BPE.**(B)** Precision-Recall Curves for seq, seq+BPP, seq+BPE, seq+BPP+BPE.

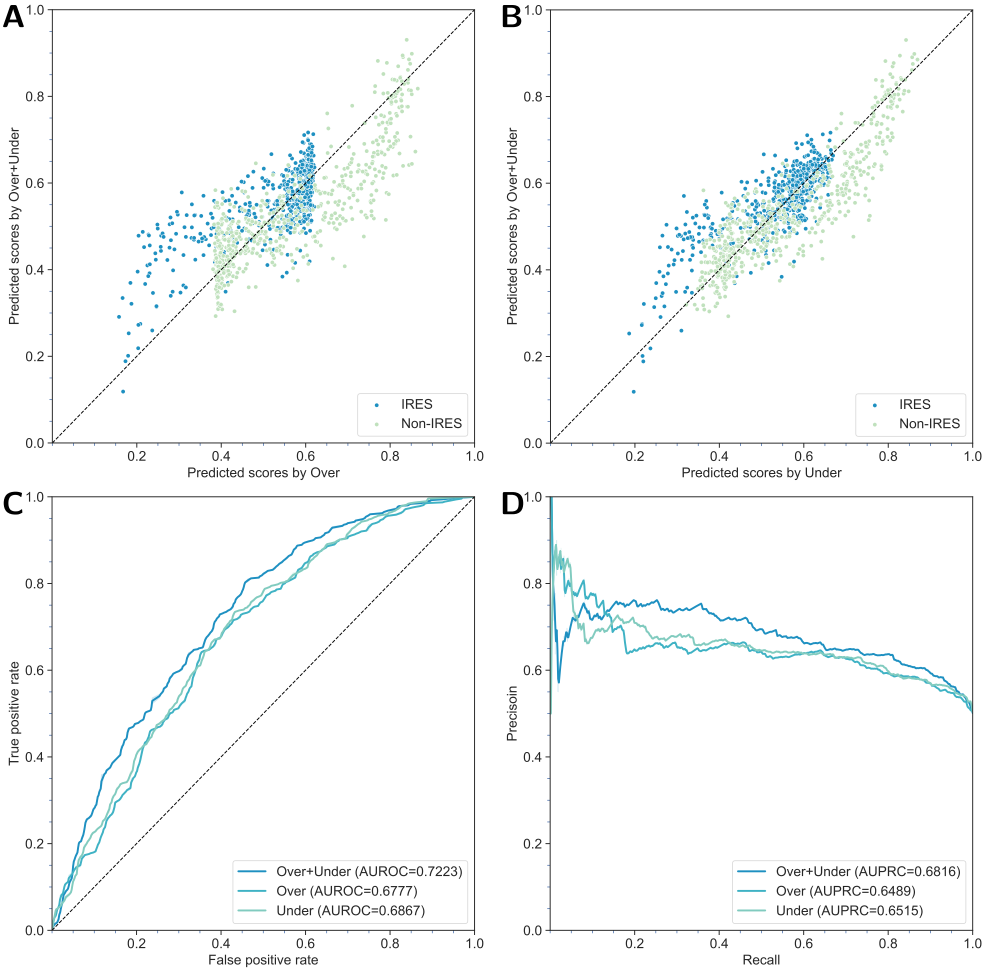

Supplementary Fig. 3: **Ablation study of IRESeek on sampling strategies for imbalanced classification.** **(A)** Head-to-head comparison of predicted scores between Over+Under and Over for IRES and non-IRES samples.**(B)** Head-to-head comparison of predicted scores between Over+Under and Under for IRES and non-IRES samples. **(C)** Receiver Operating Characteristic (ROC) Curves for Over, Under, and Over+Under Models.**(B)** Precision-Recall Curves for Over, Under, and Over+Under Models. .

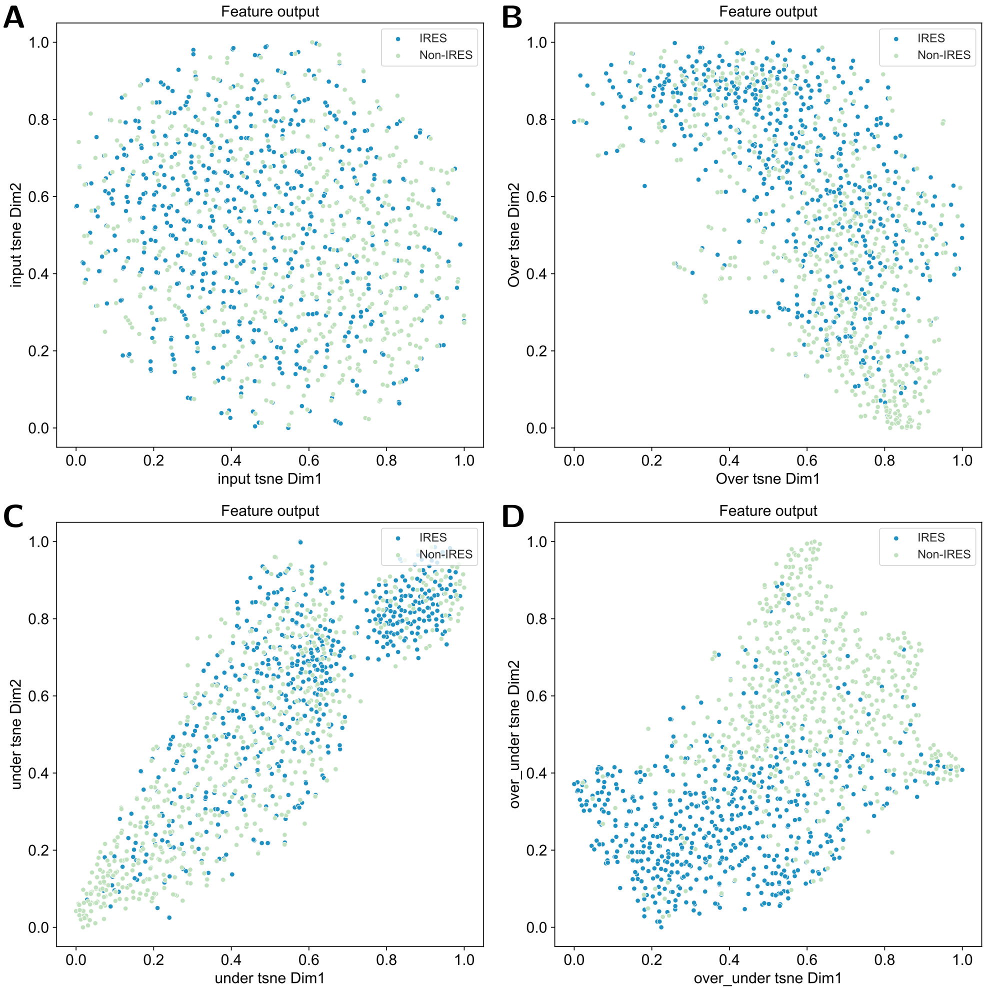

Supplementary Fig. 4: **Visualization of t-SNE results for original data and different models.** **(A)** t-SNE visualization of original input. **(B)** t-SNE visualization of the feature output of an oversampling model. **(C)** t-SNE visualization of the feature output of an undersampling model. **(D)** t-SNE visualization of the feature output of an ensemble model combining oversampling and undersampling.

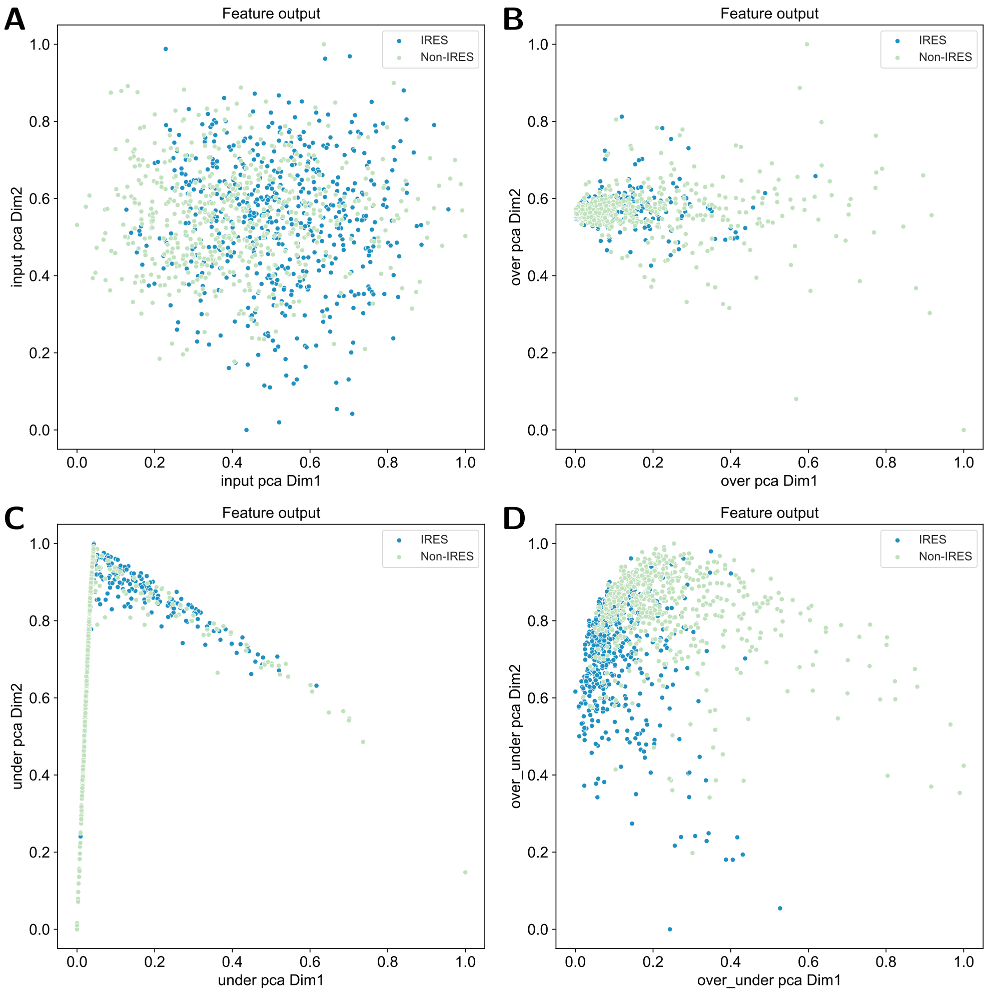

Supplementary Fig. 5: **Visualization of PCA results for original data and different models.** **(A)** PCA visualization of original input.**(B)** PCA visualization of the feature output of an oversampling model. **(C)** PCA visualization of the feature output of an undersampling model. **(D)** PCA visualization of the feature output of an ensemble model combining oversampling and undersampling.

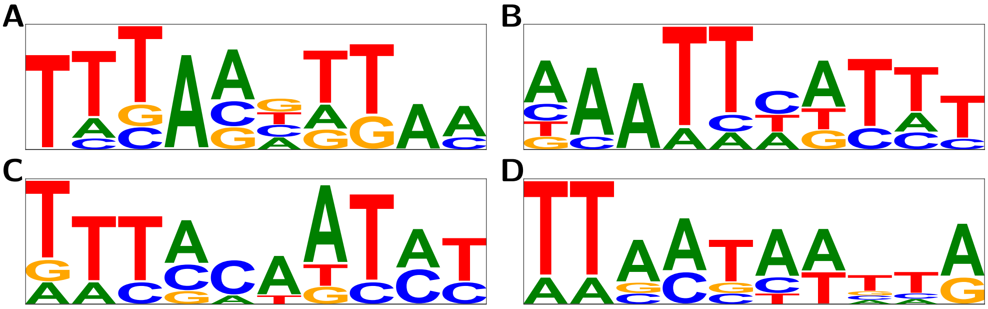

Supplementary Fig. 6: **Potential consensus motifs identified by different models (A)** Potential consensus motifs from the oversampling model.**(B,C,D)** Potential consensus motifs from the undersampling models.

2 Supplementary Tables

Supplementary Table 1: Summary of training and test datasets in our experiments.

phase	# sequences	length
train	12,983	174
test	1,164	174

Supplementary Table 2: Ablation study of IRESeek on structural guidance of base pair motif energy (BPE) and base pair motif probability (BPP).

Structural information	Accuracy	Precision	F1	MCC	AUROC	Recall
seq	0.648	0.621	0.683	0.303	0.703	0.758
seq and BPE	0.650	0.628	0.678	0.305	0.697	0.735
seq and BPP	0.650	0.626	0.681	0.306	0.707	0.746
seq and BPP,BPE	0.666	0.635	0.700	0.341	0.722	0.780

Supplementary Table 3: Ablation study of IRESeek on sampling strategies for imbalanced classification.

Sampling	Accuracy	Precision	F1	MCC	AUROC	Recall
over	0.641	0.620	0.669	0.286	0.678	0.727
under	0.651	0.629	0.678	0.307	0.687	0.735
over and under	0.666	0.635	0.700	0.341	0.722	0.780

Supplementary Table 4: The performance data of DeepIRES, IRESpy and IRESfinder

Method	Accuracy	Precision	F1	MCC	AUROC	Recall
RESfinder	0.475	0.475	0.475	-0.050	0.449	0.474
DeepIRES	0.507	0.518	0.284	0.018	0.584	0.196
DeepCIP	0.654	0.626	0.688	0.315	0.721	0.765
IRESeek	0.666	0.635	0.700	0.341	0.722	0.780

Supplementary Table 5: Performance comparison of different sequence and structure models with IRESeek

model	ACC	Precision	F1	MCC	AUROC	Recall
BiLSTM and GCN	0.623	0.600	0.664	0.255	0.667	0.743
CNN and GCN	0.626	0.603	0.663	0.257	0.673	0.735
Transformer and GCN	0.632	0.607	0.670	0.270	0.677	0.746
BiLSTM + Transformer and GCN	0.641	0.612	0.673	0.279	0.676	0.749
CNN + Transformer and GCN(Ireseek)	0.666	0.635	0.700	0.341	0.722	0.780

Supplementary Table 6: Hyperparemeter optimization for IRESeek

Hyperparameter	Values	Final value
Graph convolutional network layer	2,4,8	8
Hidden layer feature dimension	16,32,64	32
1D cnvolution layer	1,2,3	3
1D MaxPool1d kernel_size	2,3,4	3
1D convolution kernel_size	2,3,4,5	5,3,3
Transformer encoderlayer	2,4,8	8
Learning rate	2e-2,2e-3,2e-4,2e-5	0.002

Supplementary Table 7: Hardware and inference time for IRESeek

Model	GPU	GPU Memory	CUDA Version	Inference Time(ms/seq(174 nt))	
				GPU	CPU
IRESeek	NVIDIA A10	24GB	12.1	8.0	15.7
DeepCIP	NVIDIA A10	24GB	11.3	10.4	18.5
DeepIRES	NVIDIA A10	24GB	NULL	NULL	28.9
IRESfinder	NVIDIA A10	24GB	NULL	NULL	86.1

Supplementary Table 8: IRESeek Performance with diverse GCN Layers & Sampling Models.

Sampling	lay 2			lay 4			lay 8		
	Precision	Recall	MCC	Precision	Recall	MCC	Precision	Recall	MCC
Over	0.649	0.572	0.265	0.629	0.727	0.301	0.620	0.727	0.286
under	0.650	0.607	0.281	0.627	0.718	0.294	0.629	0.735	0.307
over and under	0.637	0.658	0.284	0.626	0.749	0.308	0.635	0.780	0.341

Supplementary Table 9: IRESeek Performance with diverse hidden layer feature dimension & Sampling Models.

Sampling	Dim 16			Dim 32			Dim 64		
	Precision	Recall	MCC	Precision	Recall	MCC	Precision	Recall	MCC
Over	0.644	0.603	0.270	0.620	0.727	0.286	0.594	0.838	0.290
under	0.629	0.689	0.283	0.629	0.735	0.307	0.616	0.797	0.315
over and under	0.635	0.706	0.303	0.635	0.780	0.341	0.627	0.759	0.315
